# De novo variants in the alternative exon 5 of *SCN8A* cause epileptic encephalopathy

**DOI:** 10.1038/gim.2017.100

**Published:** 2017-10-02

**Authors:** Epilepsy Genetics Initiative

**Affiliations:** 1 Columbia University Medical Center, New York, New York, USA

**Keywords:** alternative exon, epilepsy, epileptic encephalopathy, *SCN8A*, whole-exome sequencing

## Abstract

**Purpose:**

As part of the Epilepsy Genetics Initiative, we re-evaluated clinically generated exome sequence data from 54 epilepsy patients and their unaffected parents to identify molecular diagnoses not provided in the initial diagnostic interpretation.

**Methods:**

We compiled and analyzed exome sequence data from 54 genetically undiagnosed trios using a validated analysis pipeline. We evaluated the significance of the genetic findings by reanalyzing sequence data generated at Ambry Genetics, and from a number of additional case and control cohorts.

**Results:**

In 54 previously undiagnosed trios, we identified two de novo missense variants in *SCN8A* in the highly expressed alternative exon 5 A—an exon only recently added to the Consensus Coding Sequence database. One additional undiagnosed epilepsy patient harboring a de novo variant in exon 5 A was found in the Ambry Genetics cohort. Missense variants in *SCN8A* exon 5 A are extremely rare in the population, further supporting the pathogenicity of the de novo alterations identified.

**Conclusion:**

These results expand the range of *SCN8A* variants in epileptic encephalopathy patients and illustrate the necessity of ongoing reanalysis of negative exome sequences, as advances in the knowledge of disease genes and their annotations will permit new diagnoses to be made.

## Introduction

The Epilepsy Genetics Initiative (EGI) is a signature program of Citizens United for Research in Epilepsy (http://www.cureepilepsy.org/egi/index.html). It was created to house and periodically reanalyze exome sequence data for patients with seizure disorders who have had clinical exome or genome sequencing performed as part of their medical care. The EGI reanalyzes data within the repository every 6 months with two broad goals: (i) to identify novel epilepsy genes through aggregate analyses of one of the largest sources of exome sequence data in patients with epilepsy and (ii) to reanalyze exome sequence data, using the most up-to-date knowledge, for missed genetic diagnoses in epilepsy patients who initially received an inconclusive result. Newly identified genetic diagnoses are returned to patients and families through their referring physician.

Through the work of the EGI, we report here the identification of three novel disease-causing variants in alternative exon 5 A of *SCN8A* in three unrelated patients with epilepsy. These diagnoses were missed by clinical exome sequencing because, at the time of analysis, exon 5 A was not recognized as protein coding in the consensus coding sequence database (CCDS; https://www.ncbi.nlm.nih.gov/projects/CCDS/).

The *SCN8A* gene encodes the sodium channel Na_v_1.6. Mutations cause *SCN8A* epileptic encephalopathy or early infantile epileptic encephalopathy type 13 (OMIM 614558), accounting for ~1% of epileptic encephalopathy cases.^1^ *SCN8A* encephalopathy is typically associated with seizures beginning in infancy, developmental delay and varying degrees of impaired speech and motor function. Most patients have multiple seizure types, and onset is generally not associated with fever or illness.

**Figure 1:**
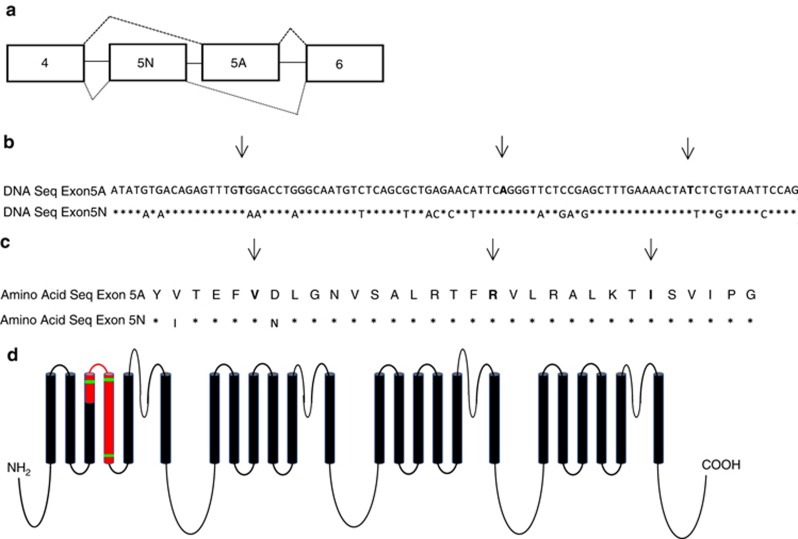
**Exon 5 of** ***SCN8A***. (**a**) Exon 5 is encoded by two sequences in the genome (exons 5 N and 5 A). Only one of the two exons remains in the transcripts after splicing occurs. The nucleotide (**b**) and amino acid sequences (**c**) of exons 5 N and 5 A are nearly identical. Asterisks indicate the sites with the same sequence and arrows highlight the sites where a novel disease-causing *SCN8A* variant was identified in exon 5 A in this study. All disease-causing variants were found at sites where the sequence was identical between exons 5 A and 5 N. (**d**) Exon 5, highlighted in red, spans two transmembrane domains and a small extracellular region of Na_v_1.6. The three disease-causing variants, marked in green, were all located in the regions encoding transmembrane-spanning portions of the protein. (Adapted from ref. 4.)

Na_v_1.6 consists of four homologous domains (DI–DIV), each of which contains six transmembrane segments (S1–S6).^2^ The *SCN8A* gene is comprised of 26 protein-coding exons.^3^ The majority of pathogenic variants in *SCN8A* are de novo missense variants or missense variants inherited from a mosaic parent.^1^ *SCN8A* contains two pairs of tandemly duplicated, mutually exclusive, alternatively spliced exons with a common evolutionary origin—exons 5 A/5 N and exons 18 A/18 N (Figure 1).^4^ Exons 5 and 18 encode portions of transmembrane segments S3 and S4 in domain I and domain III of Na_v_1.6, respectively. The 18 N transcript contains a conserved in-frame stop codon, which is predicted to result in protein truncation and is widely expressed at low levels in nonneuronal tissue.^4^ Exons 5 N (neonatal) and 5 A (adult) differ by two out of 31 amino acids (Figure 1).^5^ In humans and rodents, there is evidence that the expression of exon 5 A increases during development;^5, 6^ the expression of “neonatal” exon 5 N decreases over time, but it continues to be expressed at a low level in the adult brain. This is confirmed in exon-level data publically available in the BrainSpan database (http://www.brainspan.org/). Before this work, five disease-causing variants had been identified in exon 5 N of the *SCN8A* gene, and none had been described in exon 5A.^7^

## Materials and methods

In this study, we analyzed exome sequence data from 54 unrelated probands and their unaffected parents (trios). Sequence data were generated at GeneDx, Ambry Genetics, University of California Los Angeles, Children’s Hospital of Philadelphia, the Laboratory of Personalized Genomic Medicine at Columbia University, the Broad Institute, and the Center for Advanced Studies, Research and Development in Sardinia. We also secondarily evaluated: (i) 13,448 unrelated control samples (not ascertained for neuropsychiatric conditions) sequenced at the Institute for Genomic Medicine, (ii) individuals with nonlesional focal epilepsy (*n* = 1,187), genetic generalized epilepsy (*n* = 640), and epileptic encephalopathy (*n* = 280) who were sequenced as part of the Epi4K Consortium,^8, 9^ and (iii) 3,693 individuals who underwent diagnostic sequencing through Ambry Genetics, 1,013 of whom had been diagnosed with a seizure disorder. The study was approved by the institutional review boards at Columbia University Medical Center, University of California San Francisco, Children’s Hospital of Philadelphia, Boston Children’s Hospital, New York University Langone Medical Center, Ann & Robert H. Lurie Children’s Hospital of Chicago, and Duke University as well as the Solutions Institutional Review Board (Ambry Genetics). After informed consent was obtained, exome data files (FASTQ or BAM) generated at diagnostic exome sequencing establishments were transferred to the EGI repository at the Institute for Genomic Medicine at the Columbia University Medical Center.

For individuals 1 and 2, trio data were analyzed with a pipeline based on the Genome Analysis Toolkit best-practices protocol, as reported by Zhu et al.^10^ Trio sequence data were analyzed using an updated version of our established trio sequencing framework. SureSelect XT2 All Exon V4 and SureSelect Human All Exon 50 Mb XT kits (Agilent Technologies; Santa Clara, CA) were used for the exome capture in individual 1 and individual 2, respectively.

Individual 3 was identified through reinterrogation of clinical exome sequence data by Ambry Genetics following the identification of the two candidate variants of exon 5 A in *SCN8A* in the EGI cohort. The capture kit used by Ambry Genetics was xGen Exome Research Panel version 1.0 (Integrated DNA Technologies; Coralville, IA). This individual was subsequently enrolled in the EGI to obtain phenotype information.

Variants in exon 5 A of *SCN8A* are annotated throughout the paper based on the RefSeq identifier XM_005269075.1.

## Results

Fifty-four individuals were enrolled as trios through the EGI; most had severe epilepsies, particularly epileptic encephalopathies. Two were found to have a de novo variant in alternative exon 5 A of the *SCN8A* gene. The first individual found to carry a variant in exon 5 A of *SCN8A* (c.667A>G;p.Arg223Gly) had undergone exome sequencing in 2015. The clinical report was negative for causative variants in disease genes associated, or possibly associated, with the reported phenotype. The second individual had a different exon 5 A variant (c.632T>C;p.Val211Ala). This individual had undergone exome sequencing in 2014; while no diagnostic findings (pathogenic or likely pathogenic) had been reported, this *SCN8A* variant was reported as a de novo heterozygous intronic variant of uncertain significance. Clinical details of these two cases are shown in Table 1.

A review of 3,693 individuals who underwent diagnostic sequencing through Ambry Genetics revealed one additional de novo variant in *SCN8A* exon 5 A (c.692T>C;p.Ile231Thr). This finding was not identified at the time of the patient’s initial clinical sequencing. The exon is well sequenced on the Ambry Genetics sequencing platform. Clinical details of this case are shown in Table 1.

The XM_005269075.1 transcript contains exon 5 A of *SCN8A*. Exon 5 A was not included in CCDS 53794.1 or CCDS 44891.1 at the time the patients underwent sequencing, but it has since been included in the recent release 20 (CCDS81692.1). Since diagnostic companies often analyze only the secure annotations defined by CCDS, these variants were not called as pathogenic.

All three variants in exon 5 A are classified as probably damaging by PolyPhen-2,^11^ are absent from the Exome Aggregation Consortium and Genome Aggregation Database,^12^ and were not seen in any of the 13,448 in-house controls, despite sufficient sequence depth across both sets of controls at these sites to have called the variants (at least 90% of the 92 bases were sequenced at least 10-fold in more than 99% of the controls evaluated; average coverage across exon 42.3).

No missense variants were identified in exon 5 A of *SCN8A* in any of the other epilepsy cohorts we evaluated, including 280 individuals with epileptic encephalopathy, 640 individuals with genetic generalized epilepsy, and 1,187 individuals with nonlesional focal epilepsy. Each base of exon 5 A was sequenced at least 10-fold in these cohorts (average coverage across exon 55.6), so it is unlikely that a variant was missed.

## Discussion

After reinterrogation of existing sequence data, we report three de novo variants in exon 5 A of *SCN8A* that were not reported as pathogenic during previous diagnostic exome interpretations. Despite evidence in the literature of the exon being present and expressed in the *SCN8A* transcript, it was not included in previous CCDS releases; these mutations were therefore either not reported or reported as variants of uncertain significance. Nonetheless, we believe these variants are pathogenic for the following reasons:

1. All three reported individuals experienced seizure onset in the first year of life and global developmental delay, which are phenotypic characteristics consistent with those previously described in *SCN8A*^1, 13, 14^ (Table 1).
2. According to the American College of Medical Genetics criteria, these three variants would be classified as likely pathogenic. All three are de novo variants (both maternity and paternity confirmed; PS2), absent from controls (PM2), and have multiple lines of computational evidence (PolyPhen and SIFT) supporting a deleterious effect (PP3).^15^
3. In the second case, while the variant was classified as a variant of uncertain significance by the clinical laboratory, the ordering physician felt that it may contribute to the patient’s phenotype and introduced phenytoin to the patient’s drug regimen based on previous reports of marked benefit in the *SCN8A* patient population.^16^ This resulted in a greater than 90% reduction in seizures in the patient, who was previously refractory to many antiepileptic drugs. While not conclusive evidence of pathogenicity, this pattern is similar to that reported previously for patients with disease-causing *SCN8A* variants.
4. Each base located in the genomic coordinates corresponding to exon 5 A of *SCN8A* (hg19, chr12:52082789-52082880) was sequenced at least 10-fold in all 54 probands studied and in 13,228 unrelated controls. We observed no missense variants in any of the 13,228 controls evaluated where exon 5 A was completely sequenced at least 10-fold, nor in any of the other 120 controls where the exon was only partially sequenced. In the Exome Aggregation Consortium and Genome Aggregation Database, with aggregate variant frequencies from the data of more than 138,000 exome- and genome-sequenced individuals, each base in the exon was sequenced 10-fold in more than 90% of individuals, and only two missense variants were reported (c.653C>T;p.Ala218Val and c.644A>G;p.Asn215Ser) out of a total of nine variant sites in the exon. Assuming that all base substitutions in exon 5 A can occur, we estimate the expected rate of nonsynonymous variation in the exon to be 0.704 by taking the sum of the estimated mutability associated with each possible nonsynonymous substitution considering its trinucleotide context^8^ and dividing it by the sum of the trinucleotide mutability for all possible base substitutions across the exon. Given that we expect 70.4% of all possible substitutions to result in a nonsynonymous amino acid change, an observation of only two of the nine (22%) variant sites is unlikely to occur by chance (*P* = 0.004, binomial exact test). This overall pattern indicates that this exon is probably under purifying selection, and suggests that protein-altering genetic variation may also be associated with a disease phenotype.
5. One of the amino acid substitutions in exon 5 A described here, p.Arg223Gly, was previously identified in exon 5 N (NM_014191.2: c.667A>G) in a patient with epileptic encephalopathy.^17^ Functional analysis identified altered activity of the channel.^17^ A second amino acid substitution in exon 5 A described here, p.Val211Ala (c.632T>C), is located adjacent to the pathogenic exon 5 N variant p.Phe210Leu (NM_014191.3: c.628T>C).^18^

Since the alternative splicing of exon 5 is conserved across several of the mammalian sodium channel alpha subunit genes, including two other known epilepsy genes, we should consider the potential for missed diagnoses in those genes as well. For *SCN2A*, it is unlikely that diagnoses have been missed because both forms of the exon are annotated in the CCDS. In fact, Nakamura et al.^19^ identified a genetic variant in the *SCN2A* transcript variant 3 (NM_001040143.1), which is the neonatal (6 N) isoform (CCDS 33313.1), as well as genetic variants in transcript variant 1 (NM_021007.2), which contains the 6 A exon (CCDS 33314.1). In contrast, although *SCN1A* also contains transcripts with both neonatal (5 N) and adult (5 A) exons,^20^ only the adult exon 5 A is included in the CCDS. This suggests that disease-causing variants in exon 5 N of *SCN1A* may also be missed.

Given the developmental regulation of *SCN8A*, we considered that variation in the alternate exon 5 A may translate to a different age of onset or presentation in affected individuals. While one individual reported here had a later than average age of onset and a milder course, no consistent pattern was identified in our cohort compared with the phenotype of individuals with variants elsewhere in *SCN8A*.

In conclusion, we report three novel epilepsy diagnoses in *SCN8A*, all of which were located in an alternate copy of exon 5 (exon 5 A) and overlooked by clinical exome sequencing. These findings motivate immediate re-evaluation of the genomic sequence defining exon 5 A of *SCN8A*, particularly in patients with severe refractory subtypes of epilepsy. In addition, they motivate re-evaluation of other well-defined alternative exons in known epilepsy genes, which may detect missed diagnoses in the epilepsy patient population. The results also provide a clear demonstration of the value of deep analysis and iterative interrogation of clinical exome sequence data that were initially found to be inconclusive. This is important not only because new disease genes and new phenotypes for known genes are still being routinely discovered, but because our knowledge of gene structures and important genomic regions continues to evolve.

## Figures and Tables

**Table 1:** The Epilepsy Genetics Initiative (EGI) participant phenotype and summary of cases previously described in the literature

	**Summary of** ***SCN8A*** **EE cases previously described in the literature**^1, 13, 14^	**EGI individual 1**	**EGI individual 2**	**EGI individual 3**
Variant	—	c.667A>G (p.Arg223Gly)	c.632T>C (p.Val211Ala)	c.692T>C (p.Ile231Thr)
Inheritance	—	De novo	De novo	De novo
Sex	—	Female	Male	Male
Ethnicity	—	Paternal–European Maternal–Chinese	Paternal–European Maternal–Filipino	Paternal–European Maternal–European
Age at seizure onset	4–5 months on average	6 months	3 months	Febrile seizures at ~7–8 months; first unprovoked seizure at ~1 year of age
Seizure type(s)	Focal, tonic, clonic, myoclonic, and absence seizures reported; epileptic spasms; typically not associated with fever; convulsive or nonconvulsive status epilepticus in some	Clusters of head drops that evolved into extensor spasms	Tonic–clonic, then asymmetric supplementary motor seizures (left > right); tonic; focal dyscognitive	Febrile generalized tonic–clonic seizures, focal seizures, absence seizures
Development	Intellectual disability is common and may range from mild to severe; no speech for some; wheelchair dependence for some	Global developmental delay	Global developmental delay; never developed language	Global developmental delay; learning difficulty
Regression	In most cases, development is normal from birth to seizure onset; regression often slows or regresses following seizure onset	No	Yes; used to walk but regressed in setting of prolonged seizures	No
Electroencephalogram	Moderate to severe background slowing with focal or multifocal epileptiform discharges	Hypsarrhythmia with electroclinical spasms with electrodecrement	Left focal spikes, generalized spike/wave, background slowing and disorganization	Multifocal epileptiform discharges with background slowing
Magnetic resonance imaging	Variable degrees of generalized atrophy	Diffuse atrophy	Mild white matter volume loss; increased FLAIR hyperintensity in the left hemisphere in the acute setting of seizures	Normal
Other	Hypotonia, dystonia, ataxia, hyperreflexia, choreoathetosis	—	—	Hypotonia, ataxia
Diagnosis before exome sequencing	—	West syndrome of unknown cause	Epileptic encephalopathy of unknown cause	Epileptic encephalopathy of unknown cause
Response to treatment	Seizures refractory for many patients	Refractory to all anti- AEDs	Refractory to many AEDs; felbamate withdrawal precipitated convulsive status; addition of cannabidiol was reported to be helpful; once *SCN8A* diagnosis was made, phenytoin resulted in a >90% reduction in seizures	Responder to levetiracetam and topiramate; good seizure control since ~3 years of age; still has staring spells

AED, antiepileptic drug; EE, epileptic encephalopathy; FLAIR, fluid-attenuated inversion recovery.
